# Biocompatible quantum dot-antibody conjugate for cell imaging, targeting and fluorometric immunoassay: crosslinking, characterization and applications†

**DOI:** 10.1039/c9ra07352c

**Published:** 2019-10-15

**Authors:** Soubhagya Laxmi Sahoo, Chi-Hsien Liu, Monika Kumari, Wei-Chi Wu, Chun-Chao Wang

**Affiliations:** Department of Chemical and Materials Engineering, Chang Gung University 259, Wen-Hwa First Road, Kwei-Shan Tao-Yuan 333 Taiwan CHL@mail.cgu.edu.tw; Research Center for Chinese Herbal Medicine, Research Center for Food and Cosmetic Safety, College of Human Ecology, Chang Gung University of Science and Technology 261, Wen-Hwa First Road Taoyuan Taiwan; Department of Chemical Engineering, Ming Chi University of Technology 84, Gung-Juan Road New Taipei City Taiwan; Department of Ophthalmology, Chang Gung Memorial Hospital 5, Fu-Hsing Street Taoyuan Taiwan; College of Medicine, Chang Gung University 259, Wen-Hwa First Road Taoyuan Taiwan; Department of Medical Science, Institute of Molecular Medicine, National Tsing Hua University 101, Kuang-Fu Road Hsinchu Taiwan

## Abstract

Quantum dots (QDs) are important fluorescent probes that offer great promise for bio-imaging research due to their superior optical properties. However, QDs for live cell imaging and the tracking of cells need more investigation to simplify processing procedures, improving labeling efficiency, and reducing chronic toxicity. In this study, QDs were functionalized with bovine serum albumin (BSA) *via* a chemical linker. Anti-human immunoglobulin antibodies were oxidized by sodium periodate to create reactive aldehyde groups for a spontaneous reaction with the amine groups of BSA-modified QDs. An antibody-labeled QD bioconjugate was characterized using agarose gel electrophoresis, dynamic light scattering, and zeta potential. Using fluorescence spectroscopy, we found that the fluorescence of QDs was retained after multiple conjugation steps. The cell-labeling function of the QD bioconjugate was confirmed using an image analyzer and confocal microscopy. The QD bioconjugate specifically targeted human immunoglobulin on the membrane surface of recombinant cells. In addition, the QD bioconjugate applied in fluorometric immunoassay was effective for the quantitative analysis of human immunoglobulin in an enzyme-linked immunosorbent assay. The developed QD bioconjugate may offer a promising platform to develop biocompatible tools to label cells and quantify antibodies in the immunoassay.

## Abstract 2

A layer-by-layer covalent strategy is developed including the modification of QDs using BSA as a stabilizing agent and then anti-human immunoglobulin antibody as a targeting moiety.

## Graphical abstract

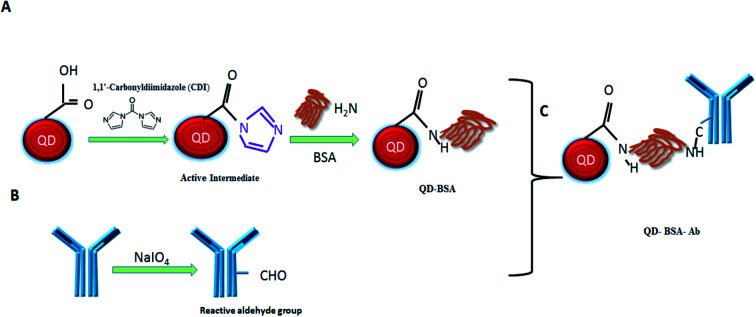

## 1 Introduction

Organic fluorophores have been successfully used in a broad variety of bio-imaging and biosensing investigations. Organic dyes with >700 nm emission suffer from low quantum yields and low photobleaching thresholds, precluding the use of intense photon beams for excitations and the possibility of long-duration cell-labeling studies.^1^ These shortcomings limit the use of organic dyes for the sensitive detection of low fluorescence targets. Semiconductor quantum dots (QDs), on the other hand, display unique fluorescence properties.^2^ These inorganic nanocrystals have been used as fluorescent probes for *in vivo* tumor imaging and detection.^3^ Because of their semiconductor core and the non-toxic shell, QDs have thermal and photochemical stability and almost no photo-oxidation.^4^ QDs have a high quantum yield, a broad emission spectrum, a narrow excitation spectrum, and outstanding resistance to photo and chemical degradation.^5^ Despite their many advantages, the cytotoxicity of QDs has been a major impediment to their biomedical application. Recently, there has been considerable concern that the natural toxic elements of the QD core (*e.g.*, cadmium/selenium, CdSe) would make the nanoparticles toxic to mammalian cells and live animals.^6^

Proteins are potentially attractive biomolecules for modifying the surface of QDs. Recent studies have shown that QDs coated with bovine serum albumin (BSA) display high stability properties.^7,8^ Thus, in this approach, BSA served as the model protein. One BSA molecule contains 60 amino acid residues and 99 carboxylic residues, and both of them could be the reactive group for the covalent linkages. Moreover, BSA displays strong binding affinity toward a variety of nanoparticles, including QDs, gold nanoparticles, and silica nanoparticles at different sites, making it a potential candidate for coating with a fluorescent probe.^9^ BSA conjugated with QDs has also been applied as an ion sensor.^10^ In addition to BSA coating, QDs are currently investigated as probes for cell labeling. Guan *et al.* prepared a transferrin-conjugated QD using multiple methods and evaluated the cell-labeling ability. The authors found that the conjugation method played a significant role in labeling the target cells.^11^ Recently, Zhang *et al.* prevent the QD's nonspecific binding to cells using ultrasonic BSA modification on QD surfaces.^12^ An efficient transfer of hydrophobic QDs from organic to aqueous BSA solution with the aid of ultrasonication can improve the QD's hydrophilicity.

Antibodies are widely used as targeting moieties with QDs for specific cell labeling. They interact with the host cell and remain adhered to the surface or internalized by endocytosis. Yang *et al.* functionalized streptavidin with QDs and biotin with antibody to form QD-antibody conjugates.^13^ Their complex procedure for antibody conjugation to a fluorescent probe may lead to the conformation changes of antibodies and reduce the antigen-recognition ability. Therefore, the surface modification of QD with target biomolecules, such as thiol groups, amino acids, and proteins, is still under exploration.^14^ Zhou *et al.* have suggested that altering the ligand types on QDs can control the energy transfer between QDs and extraneous acceptors/donors. Further, the conjugation of appropriate ligands with multi-functionality can provide the QD probes better selectivity and sensitivity.^15^ Additionally, one of the most frequently used covalent conjugation methods is *via* carbonyldiimidazole (CDI), which is a highly reactive compound with an active carbonylating agent that contains two acyl imidazole leaving groups. This crosslinker can react with a carboxylate to form an active *N*-acyl imidazole group capable of coupling with amine-containing molecules to form a stable covalent amide linkage. High-fluorescent nanoparticles with a targeting ability remain a challenge and essential need with regards to their biomedical applications.

Here, to achieve a better QD coating for cell targeting, we propose a sequential covalent strategy—the modification of CdSe/ZnS QDs using BSA as a stabilizing agent and then anti-human immunoglobulin (Ig) G antibody as a targeting moiety. BSA-modified QD (QD–BSA) was covalently linked with antibodies, termed QD bioconjugate. The surface properties of the modified QDs and QD bioconjugate were characterized by transmission electron microscope (TEM), UV-vis spectroscopy, fluorescence spectrometry, Fourier transform infrared (FTIR) spectroscopy, and zeta potential measurements. The cell-labeling efficiency of the QD bioconjugate was evaluated using a confocal laser scanning microscope in the recombinant Chinese hamster ovary (CHO) cells expressing human IgG. Finally, the developed QD bioconjugate was applied to fluoro-immunoassay for the antibody quantification using indirect enzyme-linked immunosorbent assay (ELISA).

## 2 Materials and methods

### 2.1 Materials

QDs (CdSe/ZnS, C5-0509A) were kindly donated by Dr Ray-Kuang Chiang at Far East University and Taiwan Nanocrystals Inc. (Tainan, Taiwan). BSA, CDI, sodium periodate (NaIO_4_), sodium bicarbonate, Hoechst 33342, methotrexate, trace element A and B, insulin–transferrin–sodium–selenite media supplement, trypsin–EDTA, and potassium bromide (KBr) were purchased from Sigma-Aldrich (St. Louis, MO, USA). Fetal bovine serum (FBS) was purchased from biological industries (Haemek, Israel). The bicinchoninic acid (BCA) Protein Assay Kit was purchased from Thermo Scientific (Rockford, IL, USA). The reagents for immunoassay such as affinity purified antibody to mouse IgG, peroxidase labeled antibody to mouse IgG, affinity purified antibody to human IgG, peroxidase labeled antibody to human IgG were purchased from KPL (Gaithersburg, MD, USA). Mouse IgG standard was purchased from Santa Cruz Biotechnology (Santa Cruz, CA, USA). Human IgG standard was purchased from Jackson Immuno-Research (Baltimore Pike, West Grove, PA).

### 2.2 Cell culture

CHO-K1, CHO M-CSF, and CHO βGal cells were maintained in Dulbecco's modified Eagle's (DMEM) with 10% fetal bovine serum (FBS). The hybridoma cell line (CRL-1754) secreting the mouse anti-human IgG (fragment constant) antibody, was maintained in hybridoma medium (CD Hybridoma AGT, Thermo Fisher Scientific, Waltham, MA, USA). The above-mentioned cell lines were obtained from ATCC (Manassas, VA, USA). The recombinant CHO cell line (CRL 12445) producing a monoclonal humanized antibody (IgG1) was cultured in DMEM medium containing methotrexate (200 nM), recombinant human insulin (2 μg mL^−1^), 0.1% of trace elements A, 0.1% of trace elements B and 10% of fetal bovine serum. Cells were cultured up to 90% confluence and passaged every four days with cell culture medium in a humidified atmosphere at 37 °C with 5% CO_2_. The cell growth and morphology were observed every day by a light microscope. The cell viability and cell density were determined using the trypan blue staining and Coulter Counter (Multisizer 3 model, Beckman, Brea, CA, USA). The concentration of antibody in the spent medium of CRL 1754 culture was quantified by an ELISA and the spent medium was collected for further purification described in the next section.

### 2.3 Preparation of the QD bioconjugate

The modification of the QD bioconjugate was performed according to the following protocol. Briefly, to get the stable QD bioconjugate (QD–BSA–Ab), we performed two-step experiments. In the first step, the carboxyl groups of QDs (69 μg mL^−1^) were activated by CDI linker (0.05 mM) and reacted for 30 minutes at 55 °C by vortex. Next, 3 mg mL^−1^ BSA was conjugated onto activated QDs for 2 hours at room temperature. The unconjugated products were removed by washing with distilled water using 50 kDa cutoff tubes (Vivaspin 6). The modification of BSA with QDs allows for easy attachment of another functional molecule through aldehyde groups upon activation. The anti-human IgG antibodies were purified from the spent medium of CRL 1754 hybridoma cells by using fast protein liquid chromatography (AKTAprime) and HiTrap Protein A HP column (GE, Logan, Utah, USA). In the second steps, the periodate-oxidized antibody was prepared as follows: anti-human IgG antibody (50 μg mL^−1^) was mixed with sodium periodate (0.1 mM) and reacted in dark for 30 minutes at room temperature by following the protocol from the bioconjugation textbook.^16^ For conjugation of antibodies to amine-containing molecules, the oxidized anti-human IgG antibody was dissolved with 0.2 M sodium carbonate at pH 9.6 and washed using the 50 kDa cutoff tubes to remove unreacted reagents. In the final steps, the BSA modified QD was mixed with the oxidized antibody and reacted for 2 hours to form the QD bioconjugate. The samples were washed 3 times with distilled water and stored at 4 °C. For the antibody doses effect on cell labeling, the different amounts of the oxidized antibody were conjugated with the bare QD. The stock concentration of QD bioconjugate was 69 μg mL^−1^.

### 2.4 Characterization of the QD bioconjugate

The micro-morphology of the QD samples was characterized by TEM (JM-1011, JEOL, and Tokyo, Japan) and the particle size was calculated by Image J software (version 1.43). Diluted QD sample (5 μL) was deposited on a 100-mesh copper grid (CF200-Cu, Electron Microscopy Science). The copper containing the diluted sample was dried overnight in a desiccator prior to TEM analysis. To further confirm the conjugation of protein and antibody to QDs, the molecular weight measurement was studied by using ZetaSizer® Nano ZS 90 (Malvern, Worcestershire, U.K.). A commercial BCA Protein Assay Kit was used according to the manual's instruction. BSA standards were prepared by diluting BSA from 3 to 0.03 mg mL^−1^. The samples (BSA modified QD) were mixed with the kit solutions and incubated at 37 °C for 25 minutes. Finally, the absorbance was measured at 562 nm in triplicate.

To quantify the dose of anti-human IgG antibody conjugated to QDs, we used an ELISA method. Briefly, a 96 well microplates (Nunc, Rochester, NY, USA) were coated with affinity purified antibody and blocked with the blocking buffer containing 3% of skim milk powder and 5% of sucrose in 100 mL phosphate buffered saline (PBS). The samples with different dilution were added to each well, and the microplate was incubated for 2 hours at room temperature. After the indicated time, the wells were washed three times and 100 μL of detection antibody conjugated with peroxidase was added to each well, and then the plates were incubated for 2 hours at room temperature. Further, the plate was washed three times and 100 μL of tetramethylbenzidine (TMB) solution was added to each well, which acted as a substrate for the peroxidase developing a blue color and was allowed to react for 20 minutes in the dark. The peroxidase reaction was stopped by adding 2 N sulfuric acid (50 μL per well) which changed the solution from blue to yellow, and the absorbance was measured at 450 nm with a microplate reader (Molecular Device, Sunnyvale, CA, USA). The titers of the antibodies from CRL 1754 and CRL 12445 cells were individually quantified by the sandwich ELISA methods using detection antibodies specific to mouse IgG and human IgG, respectively. A serially diluted mouse IgG and human antibodies were prepared separately for the standard curves. All experiments were performed in triplicate.

UV-vis spectroscopy (V-700, Jasco, Hachioji, Tokyo, Japan) were used to analyze the absorbance of QDs in the wavelength range of 400–700 nm. The emission spectra of the QDs, QD–BSA and QD bioconjugate were acquired using fluorescence spectroscopy (SpectroMax i3x multi-mode detection, Tokyo, Japan). All fluorescence spectra were collected at the room temperature. The excitation and emission wavelengths were 555 nm and 615 nm for the modified QDs and the QD bioconjugate. The QD samples were dispersed in distilled water and stored at 4 °C for 3 months. The fluorescence intensity was measured every week by fluorescence spectroscopy.

ZetaSizer® Nano ZS 90 (Malvern, Worcestershire, U.K.) was used to evaluate the surface charge property of the QDs, BSA modified QD, and QD bioconjugate. The samples were prepared at different pH (pH 3, 5, 7, 9, and 11) and the zeta potential was measured. All tests were performed in three replicates. Fourier transform infrared spectroscopy (Alpha, Bruker, Germany) was used to characterize the presence of specific functional groups in the bare QDs, BSA modified QD, and QD bioconjugate. Dried samples (0.8 mg) were mixed with KBr (IR grade, Sigma) powder (80 mg) and compressed into a thin membrane using a desktop pellet press (ICL, Garfield, NJ). The spectra of the samples were then analyzed by Bruker OPUS software. KBr pellet was prepared as the background signal.

### 2.5 Cell labeling efficiency by the QD bioconjugate

CRL 12445 cells were seeded onto 48 well plates at a density of 50 000 cells per well. To study the cell-specific labeling efficiency, the 24 h cultured cells were treated with the QD bioconjugate and incubated for 30 minutes at 4 °C. The cells were washed three times with PBS. In every well, the signals from ten fields were obtained to average as one measurement using IN Cell Analyzer 1000 (GE Healthcare, Piscataway, NJ). Therefore, the average of two measurements was used to analyze the statistical difference. The cell labeling efficiency was analyzed by the IN Cell Investigator software (version 1.5) following custom-developed protocol. The fluorescence excitation and emission wavelengths were 555 nm and 615 nm for the QD bioconjugate and 405 nm and 460 nm for Hoechst 33342.

The cell labeling efficiency of the QD bioconjugate was quantified as follows:1
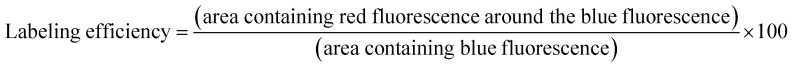


### 2.6 Specific cell targeting by the QD bioconjugate

Targeting efficiency of the QD bioconjugate was investigated by confocal microscopy. The cell lines (CHO K1, CHO M-CSF, CHO βGal, and CHO CRL 12445) were seeded on a sterile coverslip (10 mm) in 48 well culture plates. CHO M-CSF and CHO βGal cells expressed growth factor M-CSF and an enzyme β-galactosidase, respectively. CHO CRL 12445 expressed a humanized antibody against interleukin 8. The seeding cell density was maintained at 50 000 cells per well for each experiment. When the cells were 90% confluence, the live cells were incubated with the QD bioconjugate for 30 minutes at 4 °C. After the indicated incubation time, the cells were washed three times to remove the unbound QD bioconjugate with PBS and fixed by ethanol/acetic acid solution (0.5 mL 5% acetic acid in 9 mL 95% ethanol) for 20 minutes, then washed with PBS for three times. Further, the nucleus was stained with Hoechst 33342 (10 ppm) for 30 minutes and washed three times with PBS to remove excess Hoechst and unbound QDs that can interfere with the analysis. Finally, the coverslips were mounted on glass slides with DAKO fluorescent mounting media (Dako, Carpinteria, CA) and the edges of the coverslip were sealed with nail polish. Further, the confocal images were analyzed using IN Cell Investigator to determine the red fluorescence intensity. The cell labeling efficiency of the QD bioconjugate was calculated according to the eqn (1).

### 2.7 Biocompatibility studies

The biocompatibility of the QD bioconjugate was measured by standard cell counting kit-8 (CCK 8) assay (Dojindo, Rockville, MD, USA), according to the company's instructions. Briefly, cells were seeded in 96-well plates at the density of 5000 cells per well per 100 μL in duplicate. After 90% confluence, the different concentrations of the QD bioconjugate were added to cells and incubated for 1 and 4 hours at 37 °C. After the mentioned time of incubation, the medium containing the QD bioconjugate was aspirated and replaced with 100 μL of complete DMEM medium containing the 10 μL CCK 8 solution to each well and cells were incubated for additional 4 hours. Before taking a measurement, the plates were vigorously shaken for 10 minutes and samples were then read at 450 nm quantified with a microplate spectrophotometer. Untreated cells were served as control and assumed to have 100% viability. Data are presented as mean ± standard deviation. All experiments were performed at least twice at the same condition to assure reproducibility.

The cell viability was calculated as follows:
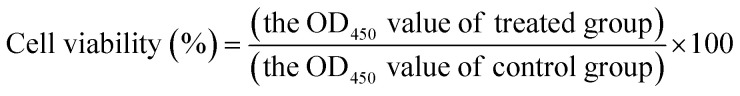


### 2.8 Fluorometric immunoassay

The black opaque microtiter plates (Nunc) were coated overnight at 4 °C with 100, 500, and 1000 ng mL^−1^ human IgG. The plates were washed with wash buffer (PBS with 0.1% tween 20) to remove excess antibody to control for non-specific binding. After removing excess antibody from well, plates were blocked with the blocking buffer (3% of skim milk powder and 5% of sucrose in 100 mL PBS) for 1 hour at room temperature. After the indicated time, the wells were washed three times with wash buffer. Then the different concentrations of the QD bioconjugate were added to the well and incubated for 2 hours at room temperature. Unbound QD-bioconjugate was removed by washing 3 times with wash buffer. The well's fluorescence was measured with *λ*_ex_/*λ*_em_ 555/615 nm by fluorescence spectroscopy.

## 3 Results and discussion

### 3.1 Functionalization of the QD bioconjugate

**Fig. 1:**
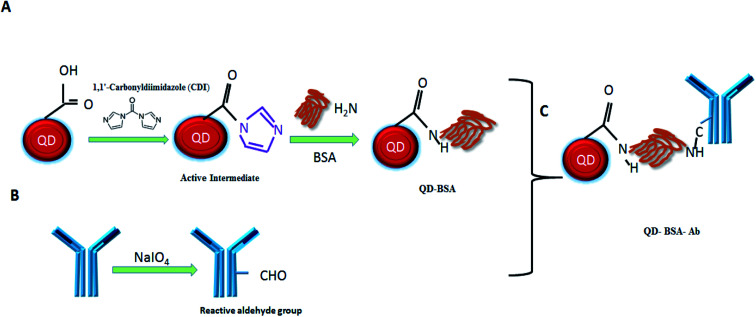
Schematic representation of the QD bioconjugate synthesis. (Step A) Carboxyl groups on QDs were activated in presence of the CDI linker. Further active carboxylate groups were reacted with amine groups on BSA to form amide bonds. (Step B) The antibody was oxidized by sodium periodate to form reactive aldehyde groups. (Step C) Finally, BSA modified QDs were conjugated with reactive aldehyde group-containing antibody to form the QD bioconjugate (QD–BSA–Ab).

The conjugation of QDs with biological molecules through the crosslinker can improve the QD biocompatibility. Accordingly, we used CDI to crosslink QDs with BSA. Briefly, this CDI linker reacted with a COOH group of QD surface to form a reactive intermediate *N*-acyl imidazole in the first step. The active intermediate was formed due to the driving force created by the liberation of carbon dioxide and imidazole. The active carboxylate further reacted with the amine groups on BSA to form amide bonds. In the second step, sodium periodate was used to oxidize the *N*-glycan of anti-human IgG antibody. Periodate oxidation is the simplest method to convert the unreactive hydroxyls into the reactive aldehyde.^17^ Finally, the oxidized antibody was conjugated with BSA-modified QDs to form the QD bioconjugate (QD–BSA–Ab). The functionalization procedure of the QD bioconjugate is shown in the schematic diagram (Fig. 1).

### 3.2 Characterization of the QD bioconjugate

**Fig. 2:**
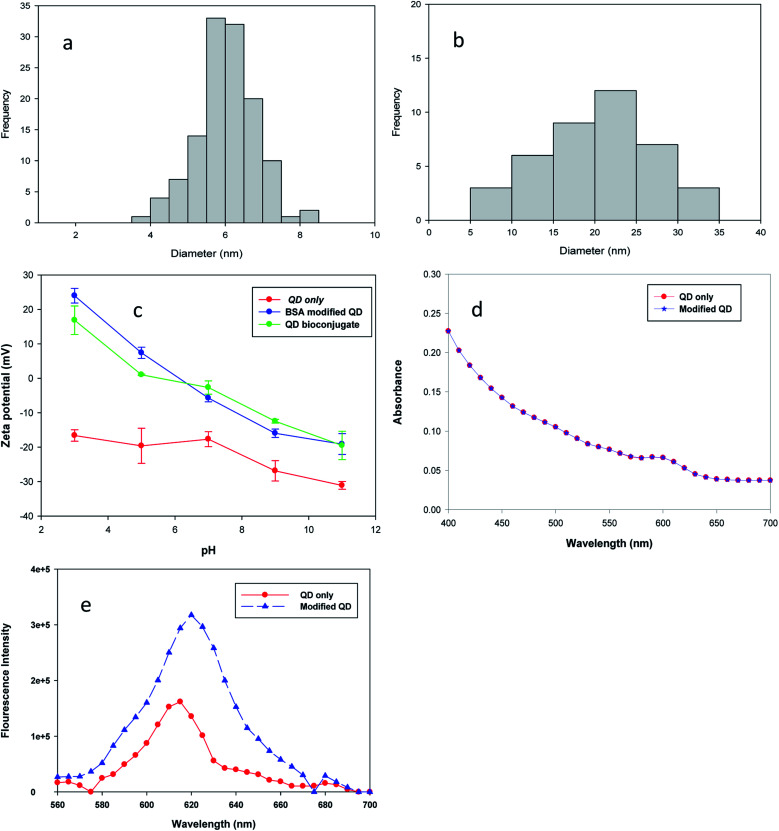
Particle size distribution histograms of bare QDs (a) and the QD bioconjugate (b). The representative histograms of diameter distribution were calculated from the data of the TEM image. Zeta potentials (c) of bare QD, BSA modified QD, and QD bioconjugate under different pH solutions. The samples were dispersed in water ranged from pH 3 to pH 11 and pH was adjusted using 0.1 N HCl and 0.1 N NaOH. UV-vis absorbance spectra (d) and fluorescence emission spectra (e) of bare QDs and BSA modified QD (excitation at 555 nm and maximal emission around 615–620 nm).

The analysis of particles' size was measured by Image J software (version 1.43), and the size distribution data were plotted in a histogram form, as shown in Fig. 2(a) and (b). Image J calculates the area, pixel value and can also create a frequency distribution against the size range of histograms.^18^ The estimated diameter of bare QDs was 6.12 nm ± 0.7 and that of the QD bioconjugate was 20.8 nm ± 5.4 by the image analysis of over 30 particles. The increasing size of the QD bioconjugate compared with bare QDs indicated that the QDs had been conjugated with the antibody. Fig. S1† shows the TEM images and particle size distribution histograms of bare QDs (a) and the QD bioconjugate (b). To further characterize these modified QDs, the molecular weights of the BSA-modified QDs and QD bioconjugate were characterized by ZetaSizer® Nano ZS 90 (Fig. S2†). The static light-scattering technique is used to measure the molecular weight from the Debye plot. The Debye plot adopts the linear relationship of *KC*/*R*_o_*P* with the protein concentration to obtain the molecular weight. The molecular weight is the inverse of the intercept. The Debye plot of the modified QDs and QD bioconjugate is used here to calculate the molecular weight. From the inverse of the intercept from the *Y*-axis (*KC*/*R*_o_*P*), the calculated molecular weights of the BSA-modified QD and QD bioconjugate were 256 and 666 kDa, respectively. *K* is a constant dependent on the sample d*n*/d*c*, *C* is the sample concentration, and *R*_o_ is the Rayleigh ratio (the ratio of scattered light intensity to incident light intensity). The molecular weight increase indicates that the modified QD was conjugated with the antibody.

Additionally, three kinds of QDs were further characterized by using a dynamic light scattering method (Zetasizer, Malvern, UK). The bare QDs had a negative surface charge as the pH ranges from 3 to 11 since they contained the COOH groups on the surfaces in Fig. 2(c). The zeta potential values of QDs, BSA modified QD, and QD bioconjugate were −17, +25, and +18 mV when the buffer was at pH 3. After conjugation of QDs with BSA, the surface charge of the QDs enhanced, this was due to the increasing number of amine group by addition of BSA molecules. The distinct positive shift of the zeta potential curve in modified QD and QD bioconjugate was ascribed to the successful modification of QDs–BSA with the antibody. Gref *et al.* suggest that the surface charge of the nanoparticle influences the coated PEG densities and its zeta potential is drastically reduced.^19^ The positive zeta potential values started to decrease for both the modified QD and QD bioconjugate as the pH was raised from 3 to 11. The zeta potential of bare QDs was observed around −32 mV at pH 11, whereas the modified QD and QD bioconjugate were shifted from −32 to −20 mV at pH 11, it indicated the successful conjugation of QDs with BSA and antibody. Our zeta potential results were similar with the previous study, where the negative surface charge of CdSe QDs was shifted to positive charge after modification with chitosan.^20^

BCA assay was performed to quantify the BSA amount in the modified QDs. The colorimetric BCA assay depends on the peptide bonds within proteins to reduce Cu^2+^ to Cu^+^. The Cu^+^ ions then chelate with two BCA molecules, producing a purple complex by measuring the absorbance at 562 nm.^21^ The BSA concentrations ranged from 30 to 3000 μg mL^−1^ in the assay. As shown in Fig. S3(a),† the BSA content in modified QDs was determined by comparing the absorbance value with a standard curve. The BSA concentration used for conjugation was 3 mg mL^−1^. The amount of BSA conjugated with QDs was estimated to be 2.2 mg mg^−1^ QDs. In addition, the quantification of anti-human IgG antibody conjugated to the modified QDs and the concentrations of antibody anti-human IgG antibody production from cell supernatant was performed by sandwich ELISA. This is a sensitive method that measures the antigen using two layers of antibodies, *i.e.*, the capture and the detection-HRP antibodies. Finally, the added substrate is converted by the enzyme to a detectable form and the absorbance intensity is detected at an absorbance of 450 nm. The tested mouse IgG ranged from 0.0156 to 2 μg mL^−1^. The concentrations of conjugated antibody in QD samples were extrapolated from the linear plot of mouse IgG standard. The estimated concentrations of antibodies conjugated with modified QDs were 18, 9, 5, and 1.1 μg mg^−1^ QDs in Fig. S3(b).† Additionally, ELISA was used to quantify the amount of humanized antibody secreted on recombinant CHO cells in Fig. S3(c).†

FTIR was used to characterize the functional groups on the modified QDs. The characteristic wavenumbers of QDs included 3438 cm^−1^ (–OH in COOH with intermolecular hydrogen bonding), 2924 cm^−1^ (symmetric CH_3_), 2855 cm^−1^ (CH_2_ stretching vibrations), and 1735 cm^−1^ (C=O), respectively. The characteristic peaks for three QD samples are shown in Fig. S4.† The amide I band represents the C=O stretching vibrations (between 1700–1650 cm^−1^) while the amide II band is associated with the N–H bending vibrations coupled with C–N stretching vibrations (between 1500–1400 cm^−1^). Comparing the FTIR spectra of the QD bioconjugate and modified QD, the characteristics peak of amide I band 1538 cm^−1^ in the modified QD had shifted to a higher wavenumber 1596 cm^−1^ in QD bioconjugate. The bands (2319 and 2855 cm^−1^) in the bare QDs were absent in the BSA modified QD. However, the amide bonds in QD bioconjugate and BSA modified QD were similar in the FTIR spectra. To verify the long-term stability of the QD bioconjugate, we measured the fluorescence intensity of the QD bioconjugate stored in the dark at 4 °C for 12 weeks. Previously, a decrease of fluorescence quantum yield of QDs is up to 50% or more, upon conjugation with biomolecules has been observed.^22^ The QD bioconjugate remained 70% of initial fluorescence after BSA and antibody conjugation up to 12 weeks of storage at 4 °C in the dark. QD bioconjugate sample was stable in long-term preservation as compared with bare QDs in Fig. S5.† The coating of the QD with BSA allows the passivation of surface states and increases the energy transfer to the core. These increase the nanomaterial's photochemical stability and resistance to photo-bleaching.^23^ Byrne *et al.* have shown that QDs are good for cell imaging studies because of their high photostability.^24^ Moreover, there were no aggregates settled onto the optical plate surface and the dispersion of the QD bioconjugate was maintained after 12 weeks storage at 4 °C. The results revealed that QD bioconjugate was stable and the surface coating of BSA enhanced the fluorescence stability of QDs.

Information on the UV-vis spectrum helps to understand the nanomaterials' characteristics, such as the absorbance, size, and concentration.^25^ The functionalization of QDs with BSA was confirmed by the overlapping of the absorption spectrum, which supported the existence of CdSe/ZnS even after BSA conjugation in Fig. 2(d). As shown in Fig. 2(e), the fluorescence intensity of QD–BSA was enhanced when the BSA was conjugated with QDs. The results are consistent with earlier researches which have reported that the fluorescence intensity of QDs increases when BSA molecules are modified on the QD surface. For example, Poderys *et al.* reported that the addition of BSA eliminates one excitation–relaxation path that increases the photoluminescence quantum yield in QDs.^26^ The increase in the fluorescence intensity of QDs should be due to a decrease in non-radiative transitions or their slow speed. Additionally, the BSA coating can lead to a decrease in the defects on the QD surface and increase the fluorescent intensity.^26^ Li *et al.* demonstrated that by increasing the BSA concentration from 0.1 to 0.5 mmol L^−1^, the fluorescence intensity of BSA-capped CdSe QD increases.^27^ The BSA coating can improve the stabilizing properties of QDs, prevent aggregation in aqueous suspension, and initiate biocompatible functionalities for further biological interactions or couplings.^28^ The fluorescence intensity of BSA-modified QDs was two times higher than that of bare QDs, as shown in Fig. 2(e). The attachment of albumin on CdTe QD results in a significant emission increase, which is attributed to the resonance energy transfer from the tryptophan moieties of albumin to CdTe nanoparticles.^23^ The fluorescence emission peak of QDs was observed at 615 nm. After QDs were modified with BSA, the maximal emission was shifted to 620 nm, owing to the size increase of the QD–BSA. This result indicated that QDs were effectively stabilized by BSA and retained their fluorescence properties using a CDI linker.

**Fig. 3:**
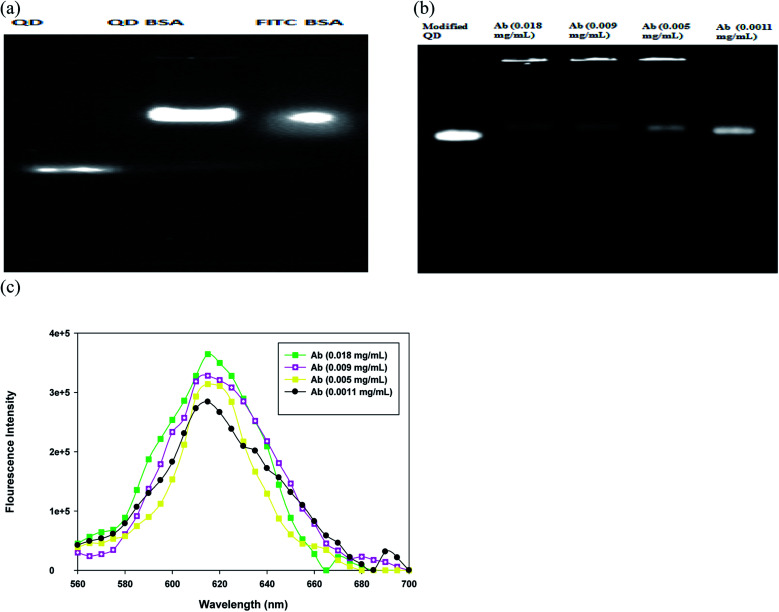
Characterization of the QD bioconjugate using agarose gel and fluorescence spectroscopy. (a) The images of agarose gel-electrophoresis of QDs, BSA modified QD and FITC BSA. (b) The images of agarose gel-electrophoresis of different antibody-loading QD bioconjugates, and their fluorescence spectra (c). The BSA concentration was fixed at 3 mg mL^−1^ in modified QD, whereas antibody loadings in QD bioconjugate were 18, 9, 5, and 1.1 μg mL^−1^. The excitation was at 555 nm.

The BSA-modified QDs and QD bioconjugate were monitored using agarose gel electrophoresis. FITC is one of the most common fluorescent tags used to conjugate proteins; its isothiocyanate group reacts with the amines group of protein to form a stable linkage. Here, the fluorescein isothiocyanate (FITC)–BSA was used as a positive control since the fluorescence of QDs and FITC can be directly detected. A strong increase in the fluorescence intensity of the QDs after the conjugation of BSA was observed in Fig. 3(a). The bare QDs exhibited a linear band in the gel and moved fast. In contrast, the QD-conjugated BSA exhibited a bright fluorescence with low mobility under the same driving voltage. The conjugation of QDs with BSA increased the overall size of QDs (Fig. 2(a and b)), which further retarded the mobility of the modified QDs (Fig. 3(a)). The data suggested that the size and surface properties contributed to the electrophoretic behavior of the modified QDs. This phenomenon is in good agreement with the theoretical predictions that the mobility in gel electrophoresis is controlled by the size and the number of charges of the particles.^29^ Bücking *et al.* studied the different cores of QDs nanoparticles conjugation with BSA and suggested that the particles' movement reduces was due to their larger size and small charge.^30^ Chan *et al.* demonstrated that in the presence of BSA, QD conjugates are well dispersed, which prevents the aggregation.^31^ We have also provided the FITC–BSA conjugated sample to compare with modified QD's fluorescence and stability. The fluorescence-based assays have a serious drawback due to their fluorescence quenching, which limits their applications.^32^ A decrease in the fluorescence of FITC–BSA may be due to the fluorescein–fluorescein quenching effects shown in Fig. 3(a). Deka *et al.* studied the effect of FITC-conjugated antibodies on cell labeling and suggested that the self-quenching effects can appear at high concentrations of FITC conjugation to antibodies.^33^ Importantly, our results provide the evidence that the BSA coating could be used to improve the fluorescence intensity of QD bioconjugate.

To optimize the concentrations of antibody linked with QD bioconjugate, four different loading of antibodies were tested in the conjugation experiment. As antibody loading in QD bioconjugate increased from 1.1 to 18 μg mL^−1^, the fluorescence intensity of the upper band increased and stuck in the well of agarose gel as shown in Fig. 3(b). The increase in antibody loading amounts increased the molecular weight of the QD bioconjugate, and in turn hindered QD's movement. However, when the antibody concentration was down to 1.1 μg mL^−1^, the fluorescence intensity of the lower band in the agarose gel clearly increased and QD bioconjugate did not stick in the well. Clearly, the QD bioconjugate incubated with 1.1 μg mL^−1^ antibody had similar mobility rate with the BSA-modified QD. The upper band (QD bioconjugate, *i.e.* BSA-modified QD plus antibody) and the lower band (BSA-modified QD) were separated on the gel because of their different mobility rates, indicating that the surface modification was successful. Our results are consistent with many observations in previous studies. For example, Yang *et al.* have demonstrated that QDs functionalized with IgG could retain the high photoluminescence. The modified nanoparticles grow bigger in size and moved slower on the gel.^13^ Additionally, Ma *et al.* have shown that by increasing the concentrations of BSA linked with QDs, the upper band shows high luminescence intensity in comparison with the lower band.^34^ Recently, Zhang *et al.* synthesize the hydrophobic QD and use the hydrophobic interaction between BSA and QDs to improve the QD's hydrophilicity.^12^ The ultrasonication-based approach is a rapid modification for the hydrophobic QDs. However, we use the hydrophilic QDs and covalent modification in the preparation of QD bioconjugate.

To further investigate the effects of antibody loading upon fluorescence of BSA-modified QD, the fluorescence characteristics of the QD bioconjugate were measured by fluorescence spectroscopy. When more antibodies were conjugated with BSA-modified QD, the fluorescence intensity increased slightly. Higher concentration of antibody (18 μg mL^−1^) enhanced the fluorescence intensity of the QD bioconjugate (Fig. 3(c)). The increase of *Q*_dot_ sensitivity has also been observed when increasing concentrations of anti-synaptophysin antibody in QD.^35^ Pang *et al.* have reported that the emission peak is slightly shifted and fluorescent intensity is enhanced when CdTe/ZnS QDs conjugated to the anti-vegf monoclonal antibody.^36^ This could be attributed to the coating of the antibody using the covalent bond. Additionally, this conjugation weakens the interaction between dipole–dipole and shortens the Stokes shifts.^36^ Our results suggested that periodate oxidized anti-human IgG antibody could also efficiently conjugated with BSA-modified QD and enhanced the fluoresce intensity of the QD bioconjugate.

### 3.3 Targeting efficiency of the QD bioconjugate

**Fig. 4:**
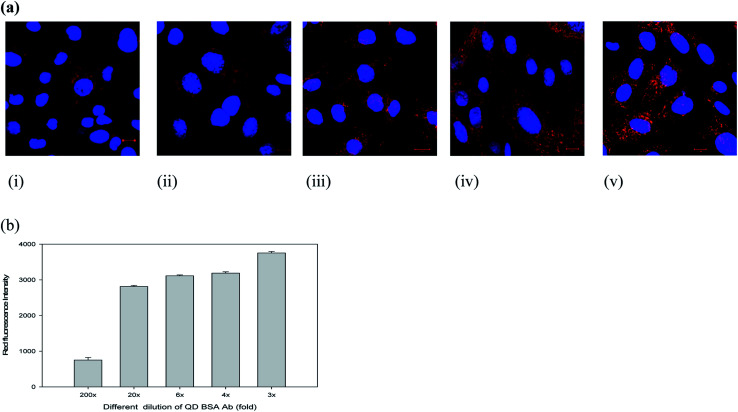
Targeting of recombinant CHO CRL 12445 cells expressing the target antigen by various concentrations of the QD bioconjugate. The confocal images (a) and the fluorescence intensity of the QD bioconjugate on cell surface antibody in recombinant CHO cells expressing the target antigen (b). Different dilution folds (i) 200×, (ii) 20×, (iii) 6×, (iv) 4×, and (v) 3× of the QD bioconjugate were incubated with two CHO cell lines for 30 minutes. Blue fluorescence indicated the nuclei stained with Hoechst and red fluorescence indicated the cell surface labeled by the QD bioconjugate. The stock concentration of QD bioconjugate was 69 μg mL^−1^. *n* = 2.

To evaluate the cell labeling function of the QD bioconjugate, recombinant Chinese hamster ovary (CHO) cells (CRL 12445) expressing the humanized antibody was chosen as a model cell line. The QD bioconjugate linked with anti-human IgG antibody was used to target this specific CHO cell. Our results demonstrated that all QD bioconjugates prepared with different antibody amounts could specifically label the cell targets. To further confirm the specific labeling ability of the QD bioconjugate, the detection of the targeted antigen on the CHO cells was observed using a confocal microscope. Fig. 4(a) showed a confocal image of recombinant CHO cell labeling by different concentrations of the QD bioconjugates prepared with 18 μg mL^−1^ of antibody. The cell membrane was clearly and evenly labeled by the QD bioconjugate in red. When more of the QD bioconjugate was added, more red dots were observed.

Different dilutions of the QD bioconjugate were incubated with CHO cells for 30 minutes and imaged by confocal microscopy in Fig. 4(a). QD bioconjugates display high labeling efficiency even at lowest concentration. At highest concentration, there is no QD aggregation. These results indicated that the modified QD bioconjugate bearing anti-human IgG antibody could bind with the humanized antibody expressed on the membrane surface of CRL 12445 cells. Moreover, the total fluorescence intensities were measured from cells incubated with different fold dilutions of the QD bioconjugates, which helps to understand quantitatively the binding specificity of the QD bioconjugate with recombinant CHO cells. As shown in Fig. 4(b), the QD fluorescence intensity of labeled cells was quantified by IN Cell Analyzer. Even if we used 20× QD bioconjugate, the antibody conjugated QDs could still recognize the specific target on cells. If the fluorescent probes are large, then they reduce the accessibility to target molecules due to their steric hindrance and slow diffusion rate.^37^ In this study, the average size of the QD bioconjugate was 19 nm, which could efficiently label the secreted antibody on recombinant CHO cells.

**Fig. 5:**
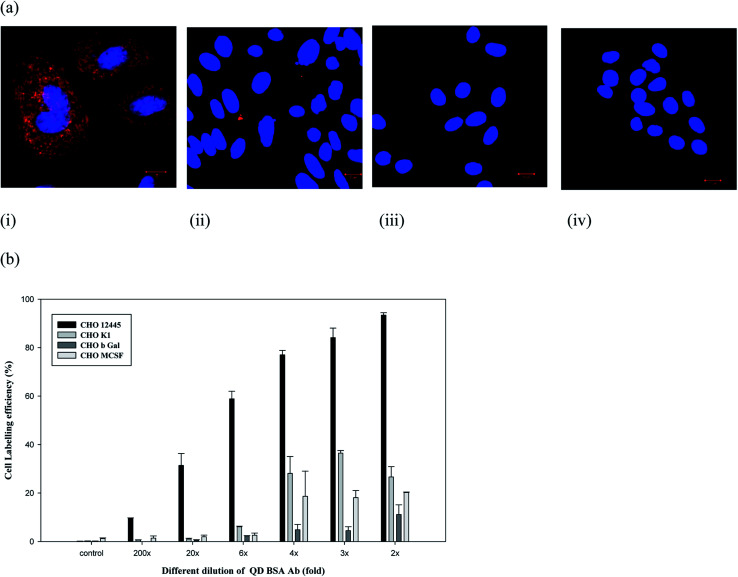
Labeling efficiency of recombinant CRL 12445 cells (i), CHO K1 (ii), CHO βGal (iii), and CHO M-CSF (iv) by the QD bioconjugate (20×) with a 30 minute staining. Confocal image (a), and specific labeling efficiency of the QD bioconjugate (2–200×) was quantified using IN Cell Analyzer (b). Blue fluorescence indicated the nuclei stained with Hoechst 33342 and red fluorescence indicated the QD bioconjugate labeled human IgG on cells surface. *n* = 2. The stock concentration of QD bioconjugate was 69 μg mL^−1^.

However, the nonspecific binding of QDs to cells has also been observed in several studies.^38,39^ To ensure the nonspecific binding of the QD bioconjugate, a labeling experiment was performed using different cell lines. The amount of nonspecific fluorescence was quite low for the negative controls, like CHO M-CSF, CHO βGal, and CHO K1 cells, as compared to the target-bearing recombinant CHO cells, specifically labeled by the QD bioconjugate (Fig. 5(a and b)). CHO K1 cells exhibited only occasional fluorescent regions indicating some QD attachment. CHO M-CSF and CHO βGal cells did not display any fluorescence, which indicated no QD attachment in Fig. 5(a). Qualitatively, the bright fluorescence labeling around the cells was not observed in this control experiment when labeled with QDs, indicating a low occurrence of nonspecific binding between cells and QDs. Therefore, the QD bioconjugate could bind the specific antigen on the plasma membrane of cells. Generally, non-specific binding is related to the low selectivity of the nanoparticles. In our study, the QD bioconjugate contains the anti-human IgG antibody as the targeting ligand, which could improve selectivity of the QDs and in turn reduce the non-specific binding to cell lines without surface markers.

### 3.4 Cell compatibility of the QD bioconjugate

**Fig. 6:**
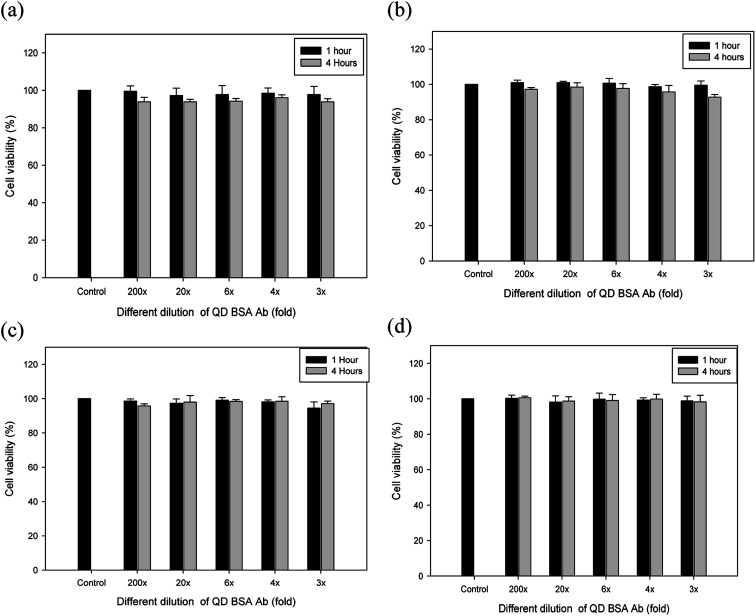
Biocompatibility of the QD bioconjugate in different cell lines CRL 12445 (a), CHO K1 (b), CHO βGal (c) and CHO M-CSF (d) cells. Measurements were performed in triplicate with error bars representing the standard deviation. The stock concentration of QD bioconjugate was 69 μg mL^−1^. *n* = 3.

The biocompatibility appears to be largely dependent upon various factors, such as size, concentrations of QDs, surface chemistry, and coating ligands. Moreover, the cytotoxicity of CdSe is related to the release of Cd ions. QD (CdSe/ZnS) with the ZnS shell or coating reduces the release of Cd ions from the particle surface and its fluorescence is also maintained for a longer period of time than the CdSe alone.^2,40^ To evaluate the dose dependency of biocompatibility of our QD bioconjugate (QD–BSA–Ab), the viability of different cells, including CRL 12445, CHO K1, CHO βGal, and CHO M-CSF cells, were determined using a standard cell viability assay. All the cell lines were incubated with different concentrations of QD bioconjugates for one and four hours, and the cell viability was measured in terms of absorbance at a wavelength of 450 nm using a UV-vis spectrophotometer. As illustrated in Fig. 6, no detectable level of cytotoxicity was observed at the different folds (3–200×) of the QD bioconjugates in four cell lines. At all QD dilutions, the cell viability was above 95% for all cell lines. Several authors demonstrated that QDs without coating is toxic to cells, but BSA coating reduces the toxicity.^41,42^ The low cytotoxicity of the QD bioconjugate may be due to the inherent properties of the BSA, such as the ion-binding capability.^43^ Thus, the cell compatibility of our QD bioconjugate was confirmed using four different cell lines.

### 3.5 Antibody density effect of the QD bioconjugate on cell labeling

**Fig. 7:**
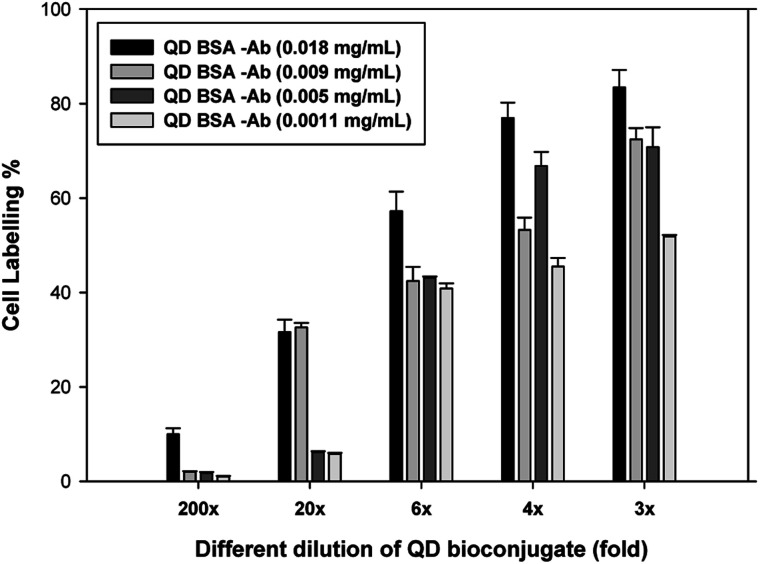
Cell labeling efficiency using different antibody-loaded QD bioconjugate. Modified QD concentration was fixed at 69 μg mL^−1^, whereas the antibody loadings in QD bioconjugates were 18, 9, 5, and 1.1 μg mL^−1^, respectively. The tested CHO CRL 12445 cells express humanized antibody that can be recognize by the antibody loaded on QD bioconjugate. The stock concentration of QD bioconjugate was 69 μg mL^−1^. *n* = 2.

We first compared the different doses of antibody such as 18, 9, 5, and 1.1 μg mL^−1^ conjugated to the modified QD in Fig. 7. Different dilutions (200-, 20-, 6-, 4-, and 3-fold) of the QD bioconjugate were incubated with live recombinant CHO cells for 30 minutes and washed 3 times with PBS. The cell labeling efficiency was increased with increasing concentrations of the QD bioconjugate. All the concentrations had showed good labeling efficiency still after several wash steps. The 2-fold dilution of the QD bioconjugate containing 18 μg mL^−1^ of antibody could efficiently label 88% of recombinant CHO cells and the 200-fold dilution could also label 10% of cells. After being washed several times, the QD bioconjugate could retain the ability to label the cells at lower fold of dilution. Whereas, the QD bioconjugate containing 9 μg mL^−1^ of antibody at 3-, 4-, 6-, 20-, and 200-fold dilution could label 75, 50, 40, 35, 5% of the targeting cells. The QD bioconjugate containing low concentrations of antibody such as 5 and 1.1 μg mL^−1^ showed low cell labeling efficiency in CHO cells. The amount of Ab conjugated on QDs surface affected the labeling efficiency. The optimization of antibody loading was required to achieve efficient cellular labeling. We finally chose QD bioconjugate containing 18 μg mL^−1^ of antibody for the further labeling experiment. Chalmers *et al.* have suggested that several antibody molecules can conjugates per QD, so it requires higher protein concentrations to achieve optimal cellular resolution.^44^ We found that QD bioconjugate could efficiently label the targeting cells. To prove that QD bioconjugate could properly label the recombinant CHO cells, the images of different cells were observed using the confocal microscopy in the next section. Using this technique, we can not only evaluate the labeling ability but also test the detection of the targeted antigen on the CHO cells by using the QD bioconjugate.

### 3.6 QD bioconjugate applied in fluorometric immunoassay

**Fig. 8:**
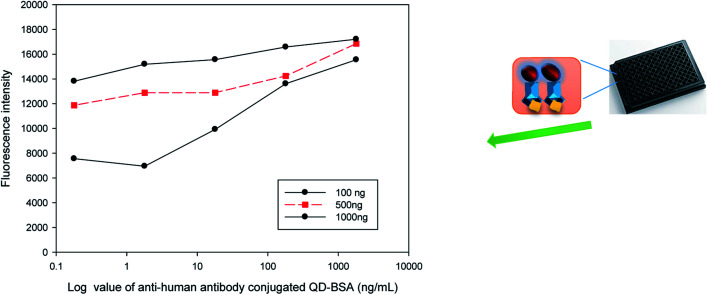
Indirect ELISA detection of human IgG (100, 500, 1000 ng per well) by the QD bioconjugate containing anti-human IgG antibody. Varying concentrations (0.18–1800 ng mL^−1^) of the QD bioconjugate were used to detect the human antibody coated on the black plate the QD bioconjugate concentration in the *x*-axis was on the log scale.

The feasibility of a QD bioconjugate as a sensitive immuno-fluorescent agent for indirect ELISA analysis was evaluated in this study. Fig. 8 indicates that the fluorescence intensity was related to the concentrations of the antigen. More antigens coated on the plate captured more QD bioconjugate and had stronger fluorescence. The results indicated that anti-human IgG antibody-conjugated QDs could specifically label the target (human IgG) *via* the antibody–antigen binding. The antigen concentration is one of the most important parameters that would affect the response and sensitivity of the immuno-sensor. Different amounts (100, 500, and 1000 ng per well) of human IgG were coated separately on a 96-well microtiter plate. Various concentrations of the QD bioconjugate (containing anti-human IgG antibody), *i.e.*, 0.18, 1.8, 18, 180, and 1800 ng mL^−1^, were added to detect the human IgG on the plate surface. The fluorescence intensity increased when the concentrations of the QD bioconjugate increased from 0.18 to 180 ng mL^−1^ per well, and it reached saturation when over 180 ng mL^−1^. This was due to all available moieties of human IgG being occupied with the QD bioconjugate. Thus, we observed that the cell's fluorescence increased by the increase of IgG concentration in the QD bioconjugate. Human IgG doses, such as 100 and 500 ng per well, were more sensitive to the QD bioconjugate. Several recent immunoassays have begun to have improved sensitivities in the detection using QDs.^45,46^ In this study, the detection of 180 g mL^−1^ antigen and a broader range (0.18–1800 ng mL^−1^) were obtained using the QD bioconjugate.

## 4 Conclusions

In this study, the conjugation of QDs to BSA used CDI as a linker and the moiety of anti-human IgG antibody could increase the specific targeting of the QD bioconjugates. Our method utilized a convenient strategy to sequentially conjugate BSA and antibody on the QDs. The antibody on the QD bioconjugate retained the antigen-binding capacity and could target the antigen on both live and fixed cells. Moreover, the cell viability results demonstrated that the QD bioconjugate was non-toxic and cell compatible. Finally, the developed QD bioconjugate could be applied to a one-step and highly sensitive immuno-fluorescent assay, which halved the analysis time compared to traditional ELISA.

## Conflicts of interest

There are no conflicts to declare.

## Supplementary Material

RA-009-C9RA07352C-s001
