# Supplementary material for: Biocompatible quantum dot-antibody conjugate for cell imaging, targeting and fluorometric immunoassay: crosslinking, characterization and applications
Source: RSC Adv. 2019 Oct 15;9(56):32791–803. doi: 10.1039/c9ra07352c (PMC9073096; doi:10.1039/c9ra07352c)
Supplement: RA-009-C9RA07352C-s001 [file RA-009-C9RA07352C-s001.pdf]

Electronic Supplementary Material (ESI) for RSC Advances.
This journal is © The Royal Society of Chemistry 2019

Supplementary Information

Characterization of the QD bioconjugate

(a)

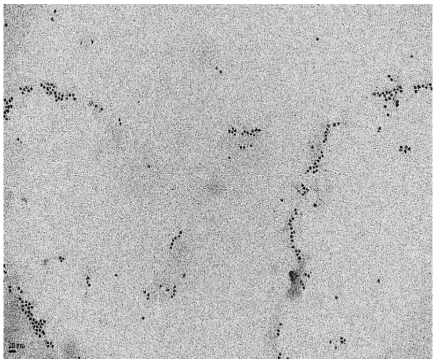

(b)

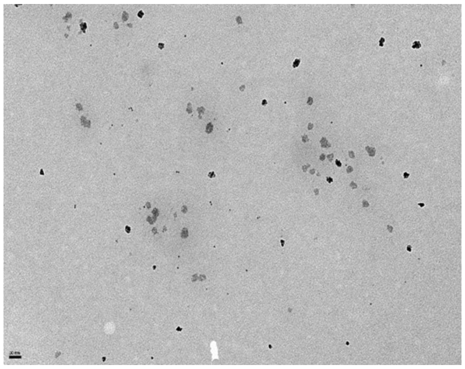

Fig. S1. TEM images and particle size distribution histograms of bare QDs (a) and the QD bioconjugate (b). The scale bars in TEM are 20 nm.

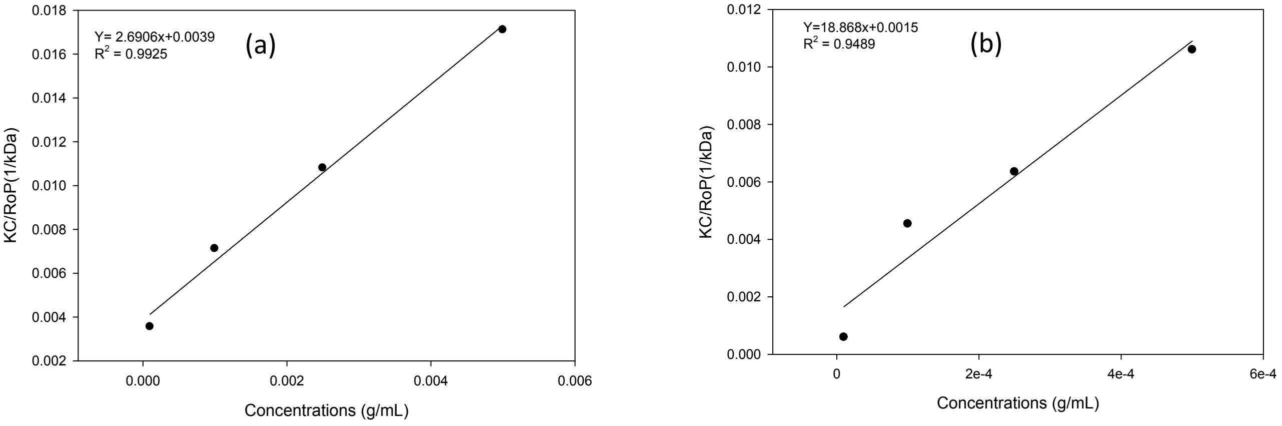

Fig. S2. Debye plot of BSA modified QD (a), and QD bioconjugate (b) for the determination of their molecular weight.

(a)

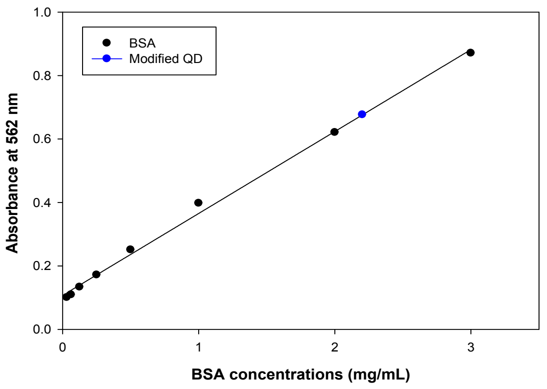

(b)

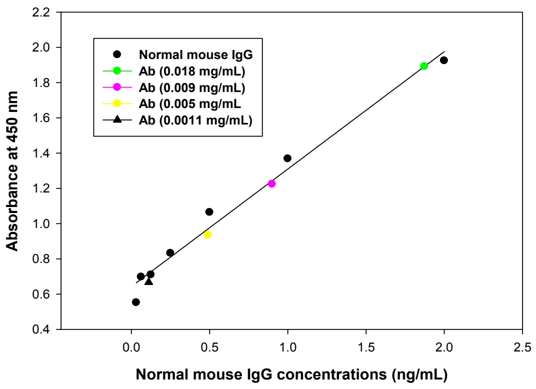

(c)

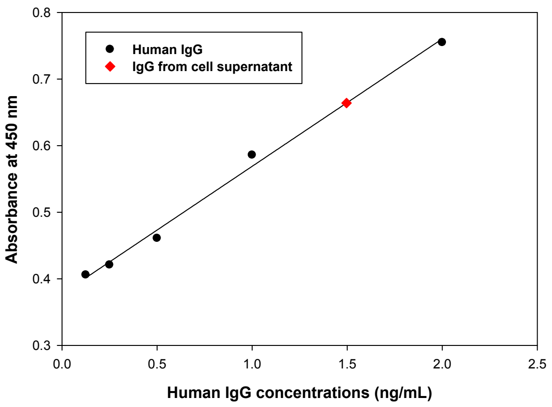

Fig. S3. Quantitative estimation of BSA and antibody content in modified QDs. (a) BCA assay for BSA modified QD (b) ELISA Assay of anti-human antibody conjugated to QD bioconjugate (c) ELISA assay for humanized antibody produced from recombinant CHO cells.

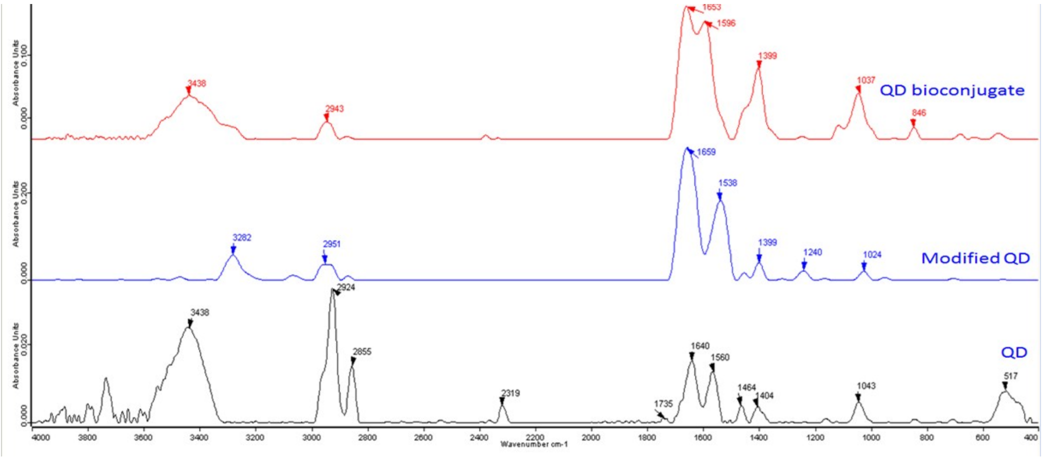

Fig. S4. FTIR spectra of the QD bioconjugate. Bare QDs (black), modified QD (blue), and QD bioconjugate (red).

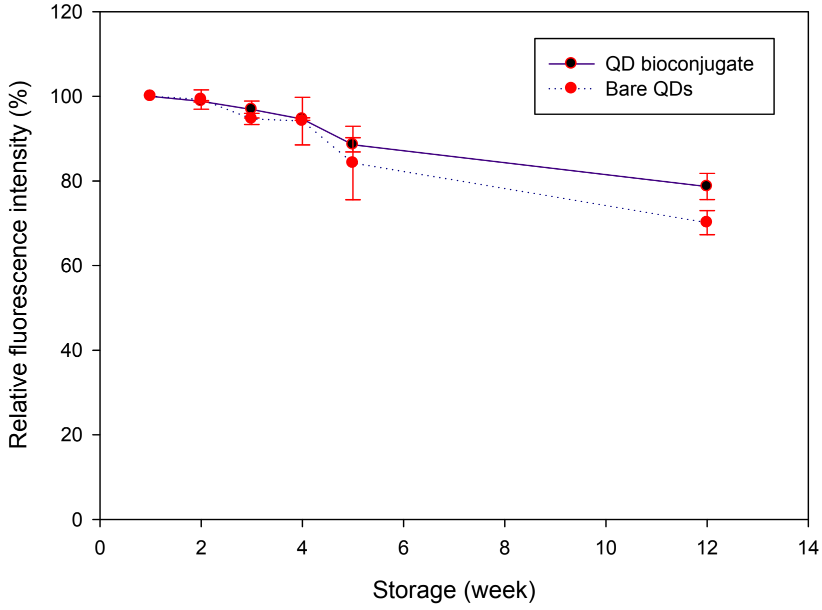

Fig. S5. Storage stability of the QD bioconjugate and QDs in the dark at 4° C.
